# Decacarbonyl-1κ^3^ *C*,2κ^3^ *C*,3κ^4^ *C*-bis­[tris­(3-chloro­phen­yl)phosphine]-1κ*P*,2κ*P*-*triangulo*-triruthenium(0) monohydrate

**DOI:** 10.1107/S1600536810013838

**Published:** 2010-04-28

**Authors:** Omar bin Shawkataly, Mohd. Aslam A. Pankhi, Chin Sing Yeap, Hoong-Kun Fun

**Affiliations:** a Chemical Sciences Programme, School of Distance Education, Universiti Sains Malaysia, 11800 USM, Penang, Malaysia; b X-ray Crystallography Unit, School of Physics, Universiti Sains Malaysia, 11800 USM, Penang, Malaysia

## Abstract

The asymmetric unit of the title *triangulo*-triruthenium compound, [Ru_3_(C_18_H_12_Cl_3_P)_2_(CO)_10_]·H_2_O, consists of one *triangulo*-triruthenium complex and one disordered water solvent molecule. Two of the 3-chloro­phenyl rings are disordered over two positions with refined site occupancies of 0.671 (3)/0.329 (3) and 0.628 (3)/0.372 (3). The water mol­ecule is disordered over two positions with refined site occupancies of 0.523 (7) and 0.477 (7). Two equatorial carbonyl groups have been substituted by the two monodentate phosphine ligands, leaving one equatorial and two axial carbonyl substituents on the two Ru atoms. The remaining Ru atom carries two equatorial and two axial terminal carbonyl ligands. In the crystal structure, mol­ecules are linked into columns along the *a* axis by inter­molecular C—H⋯Cl and C—H⋯O hydrogen bonds. The mol­ecular structure is stabilized by weak intra­molecular C—H⋯O hydrogen bonds.

## Related literature

For related structures, see: Bruce *et al.* (1988*a* ▶,*b* ▶); Chin-Choy *et al.* (1988 ▶). For the synthesis, see: Bruce *et al.* (1987 ▶). For the stability of the temperature controller used for the data collection, see: Cosier & Glazer (1986 ▶).
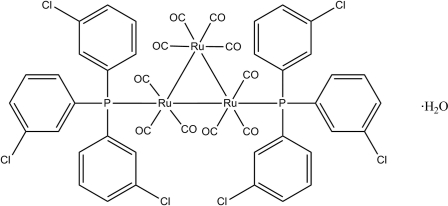


## Experimental

### 

#### Crystal data

[Ru_3_(C_18_H_12_Cl_3_P)_2_(CO)_10_]·H_2_O
*M* *_r_* = 1332.52
Triclinic, 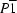

*a* = 10.8063 (1) Å
*b* = 10.9841 (1) Å
*c* = 21.3087 (2) Å
α = 76.543 (1)°
β = 89.766 (1)°
γ = 89.394 (1)°
*V* = 2459.71 (4) Å^3^
*Z* = 2
Mo *K*α radiation
μ = 1.36 mm^−1^
*T* = 100 K
0.32 × 0.17 × 0.06 mm

#### Data collection

Bruker SMART APEXII CCD area-detector diffractometer
Absorption correction: multi-scan (*SADABS*; Bruker, 2009 ▶) *T*_min_ = 0.667, *T*_max_ = 0.919
76122 measured reflections
17915 independent reflections
13849 reflections with *I* > 2σ(*I*)
*R*_int_ = 0.037

#### Refinement

*R*[*F* ^2^ > 2σ(*F* ^2^)] = 0.044
*wR*(*F* ^2^) = 0.132
*S* = 1.07
17915 reflections
735 parameters
301 restraints
H-atom parameters constrained
Δρ_max_ = 3.29 e Å^−3^
Δρ_min_ = −1.17 e Å^−3^

### 

Data collection: *APEX2* (Bruker, 2009 ▶); cell refinement: *SAINT* (Bruker, 2009 ▶); data reduction: *SAINT*; program(s) used to solve structure: *SHELXTL* (Sheldrick, 2008 ▶); program(s) used to refine structure: *SHELXTL*; molecular graphics: *SHELXTL*; software used to prepare material for publication: *SHELXTL* and *PLATON* (Spek, 2009 ▶).

## Supplementary Material

Crystal structure: contains datablocks global, I. DOI: 10.1107/S1600536810013838/sj2763sup1.cif

Structure factors: contains datablocks I. DOI: 10.1107/S1600536810013838/sj2763Isup2.hkl

Additional supplementary materials:  crystallographic information; 3D view; checkCIF report

## Figures and Tables

**Table 1:** Hydrogen-bond geometry (Å, °)

*D*—H⋯*A*	*D*—H	H⋯*A*	*D*⋯*A*	*D*—H⋯*A*
C4—H4*A*⋯Cl2^i^	0.93	2.81	3.607 (4)	145
C6—H6*A*⋯O7	0.93	2.55	3.259 (5)	134
C11—H11*A*⋯O8^ii^	0.93	2.57	3.234 (4)	129
C24—H24*A*⋯O9	0.93	2.55	3.425 (5)	157

Symmetry codes: (i) 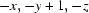; (ii) 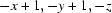.

**Fig. 1.**
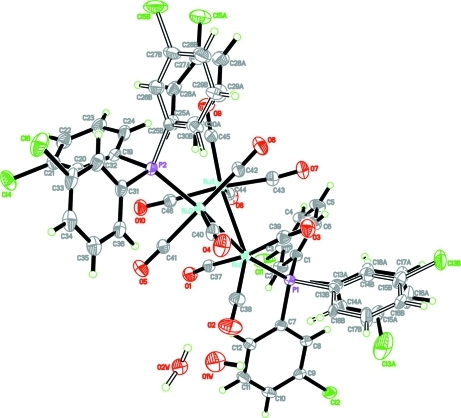
The molecular structure of the title compound with 50% probability ellipsoids for non-H atoms.

**Fig. 2.**
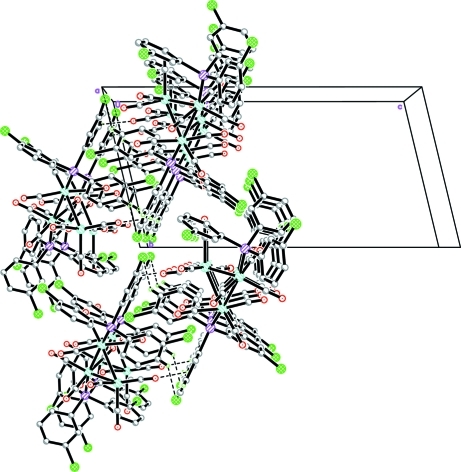
The crystal packing of (I), viewed down the a axis, showing the molecules linked into columns along a axis. Hydrogen atoms not involved in the hydrogen-bonding (dashed lines) and the solvent molecules have been omitted for clarity.

## supplementary crystallographic information

### Comment

Syntheses and structures of substituted *triangulo*-triruthenium clusters have been of interest to researchers due to observed structural variations and their potential catalytic activity. As part of our ongoing studies of phosphine substituted *triangulo*-triruthenium clusters, we report herein the structure of title compound (I).

The asymmetric unit of title compound (I) consists of one *triangulo*-triruthenium complex and one disordered water solvent (Fig. 1). The geometric parameters of title compound are comparable to those found in a related structure (Chin-Choy *et al.*, 1988). Two monodentate phosphine ligands have replaced carbonyl groups on the Ru1 and Ru2 atoms in the equatorial plane of the Ru_3_ triangle. The two ligands are approximately trans to the same Ru–Ru bond with P1–Ru1–Ru3 and P2–Ru2–Ru1 bond angles of 166.40 (2) and 167.38 (2)°, respectively. The P1–Ru1–Ru2–P2 torsion angle is 103.95 (14)°. Unlike the monosubsubstituted complexes of the type Ru_3_(CO)_11_L [where L = PPh(OMe)_2_, P(OCH_2_CF_3_)_3_] (Bruce *et al.*, 1988b), there is no pronounced difference in the Ru–Ru separations that can be correlated to the presence of the ligand. The Ru–Ru separations are in the range 2.8517 (3) to 2.8645 (3) Å. The differences in the two Ru–P separations are almost identical with those in the analogous complexes synthesized by Bruce *et al.* (1988a). In this complex, the equatorial CO groups are more inclined to preserve linearity, equatorial Ru–C–O angles averaging 176.3° while the average value of the axial Ru–C–O groups is 173.8°.

In the crystal structure, the molecules are linked into columns along the *a* axis by intermolecular C4—H4A···Cl2 and C11—H11A···O8 hydrogen bonds (Fig. 2, Table 1). The molecular structure is stabilized by weak intramolecular C6—H6A···O7 and C24—H24A···O9 hydrogen bonds (Table 1).

### Experimental

All the manipulations were performed under a dry oxygen-free nitrogen atmosphere using standard Schlenk techniques. THF was dried over sodium wire and freshly distilled from sodium benzophenone ketyl solution. The title compound (I) was prepared by mixing Ru_3_(CO)_12_ (Aldrich) and P(3-Cl-C_6_H_4_)_3_ (Maybridge) in a 1:2 molar ratio in THF at 40 °C. About 0.2 ml of diphenylketyl radical anion initiator (synthesized as per the method of Bruce *et al.*,  1987) was introduced into the reaction mixture under a current of nitrogen. After 20 min. of stirring, the solvent was removed under vacuum. Separation of the product in the pure form was done by preparative thin layer chromatography (mobile phase, dichloromethane: hexane, 1:3) and major red band was separated. Spectroscopic analyses, IR (cyclohexane): ν(CO) 2081, 2059, 2041, 2028, and 2002 cm^-1^. ^1^H-NMR (CDCl_3_, δ); 7.23-7.55 (m, aromatic protons). Single crystals of I (m.p. 159–162° C) were grown by slow diffusion from dichloromethane: methanol  at 10 °C

### Refinement

All hydrogen atoms were positioned geometrically and refined using a riding model with C–H = 0.93 Å, *U*_iso_(H) = 1.2 *U*_eq_(C) and O–H = 0.85 Å, *U*_iso_(H) = 1.5 *U*_eq_(O). Two of the 3-chlorophenyl rings (Cl3/C13–C18 and Cl5/C25–C30) are disordered over two positions with refined site-occupancies of 0.671 (3)/0.329 (3) and 0.628 (3)/0.372 (3), respectively.  These disordered benzene rings were subjected to rigid bond and similarity restraints. The same U_ij_ parameters were used for the atom pairs C15A/C15B. The water molecule is also disordered over two positions with refined site-occupancies of 0.523 (7) and 0.477 (7). The O atom of the disordered water molecule was refined isotropically. The maximum and minimum residual electron density peaks were located 1.79 Å and 0.39 Å from the H1WA and Cl6 atoms, respectively.

### Crystal data

**Table:**

[Ru_3_(C_18_H_12_Cl_3_P)_2_(CO)_10_]·H_2_O	*Z* = 2
*M**_r_* = 1332.52	*F*(000) = 1308
Triclinic, *P*1	*D*_x_ = 1.799 Mg m^−^^3^
Hall symbol: -P 1	Mo *K*α radiation, λ = 0.71073 Å
*a* = 10.8063 (1) Å	Cell parameters from 9887 reflections
*b* = 10.9841 (1) Å	θ = 2.3–32.5°
*c* = 21.3087 (2) Å	µ = 1.36 mm^−^^1^
α = 76.543 (1)°	*T* = 100 K
β = 89.766 (1)°	Plate, brown
γ = 89.394 (1)°	0.32 × 0.17 × 0.06 mm
*V* = 2459.71 (4) Å^3^	

### Data collection

**Table:**

Bruker SMART APEXII CCD area-detector diffractometer	17915 independent reflections
Radiation source: fine-focus sealed tube	13849 reflections with *I* > 2σ(*I*)
graphite	*R*_int_ = 0.037
φ and ω scans	θ_max_ = 32.7°, θ_min_ = 2.1°
Absorption correction: multi-scan (*SADABS*; Bruker, 2009)	*h* = −15→16
*T*_min_ = 0.667, *T*_max_ = 0.919	*k* = −16→16
76122 measured reflections	*l* = −32→32

### Refinement

**Table:**

Refinement on *F*^2^	Primary atom site location: structure-invariant direct methods
Least-squares matrix: full	Secondary atom site location: difference Fourier map
*R*[*F*^2^ > 2σ(*F*^2^)] = 0.044	Hydrogen site location: inferred from neighbouring sites
*wR*(*F*^2^) = 0.132	H-atom parameters constrained
*S* = 1.07	*w* = 1/[σ^2^(*F*_o_^2^) + (0.0658*P*)^2^ + 3.5923*P*] where *P* = (*F*_o_^2^ + 2*F*_c_^2^)/3
17915 reflections	(Δ/σ)_max_ = 0.001
735 parameters	Δρ_max_ = 3.29 e Å^−^^3^
301 restraints	Δρ_min_ = −1.17 e Å^−^^3^

### Special details

**Table:**

Experimental. The crystal was placed in the cold stream of an Oxford Cryosystems Cobra open-flow nitrogen cryostat (Cosier & Glazer, 1986) operating at 100.0 (1) K.
Geometry. All esds (except the esd in the dihedral angle between two l.s. planes) are estimated using the full covariance matrix. The cell esds are taken into account individually in the estimation of esds in distances, angles and torsion angles; correlations between esds in cell parameters are only used when they are defined by crystal symmetry. An approximate (isotropic) treatment of cell esds is used for estimating esds involving l.s. planes.
Refinement. Refinement of *F*^2^ against ALL reflections. The weighted *R*-factor wR and goodness of fit *S* are based on *F*^2^, conventional *R*-factors *R* are based on *F*, with *F* set to zero for negative *F*^2^. The threshold expression of *F*^2^ > σ(*F*^2^) is used only for calculating *R*-factors(gt) etc. and is not relevant to the choice of reflections for refinement. *R*-factors based on *F*^2^ are statistically about twice as large as those based on *F*, and *R*- factors based on ALL data will be even larger.

### Fractional atomic coordinates and isotropic or equivalent isotropic displacement parameters (Å^2^)

**Table:**

	*x*	*y*	*z*	*U*_iso_*/*U*_eq_	Occ. (<1)
Ru1	0.45268 (2)	0.36474 (2)	0.214149 (11)	0.01749 (5)	
Ru2	0.60936 (2)	0.17230 (2)	0.286321 (11)	0.01872 (6)	
Ru3	0.44475 (2)	0.11772 (2)	0.191744 (12)	0.02019 (6)	
Cl1	0.16663 (12)	0.42852 (9)	−0.07879 (5)	0.0476 (3)	
Cl2	0.14701 (8)	0.93629 (7)	0.00295 (4)	0.02769 (15)	
Cl4	1.13649 (9)	−0.06793 (10)	0.16900 (5)	0.0418 (2)	
Cl6	1.16192 (10)	−0.12800 (10)	0.47584 (4)	0.0423 (2)	
P1	0.28728 (7)	0.48755 (7)	0.16272 (4)	0.01911 (14)	
P2	0.75116 (8)	0.00875 (7)	0.32315 (4)	0.02300 (15)	
O1	0.5710 (2)	0.3911 (2)	0.08039 (11)	0.0309 (5)	
O2	0.6057 (3)	0.5704 (3)	0.24798 (15)	0.0492 (8)	
O3	0.3192 (2)	0.3164 (3)	0.34537 (11)	0.0330 (5)	
O4	0.6561 (3)	0.2972 (3)	0.39650 (13)	0.0413 (7)	
O5	0.8051 (2)	0.3200 (2)	0.19454 (12)	0.0319 (5)	
O6	0.4048 (2)	0.0190 (3)	0.36790 (12)	0.0350 (6)	
O7	0.2023 (2)	0.1273 (3)	0.26728 (12)	0.0308 (5)	
O8	0.3188 (2)	0.1585 (3)	0.06025 (12)	0.0340 (5)	
O9	0.4278 (3)	−0.1672 (3)	0.22420 (19)	0.0513 (9)	
O10	0.6986 (2)	0.1230 (2)	0.12431 (12)	0.0322 (5)	
C1	0.1917 (3)	0.4178 (3)	0.10969 (15)	0.0229 (6)	
C2	0.2085 (3)	0.4516 (3)	0.04299 (16)	0.0264 (6)	
H2A	0.2638	0.5143	0.0247	0.032*	
C3	0.1416 (4)	0.3905 (3)	0.00433 (18)	0.0319 (7)	
C4	0.0565 (4)	0.3000 (3)	0.0293 (2)	0.0396 (9)	
H4A	0.0110	0.2622	0.0022	0.048*	
C5	0.0396 (3)	0.2659 (3)	0.0955 (2)	0.0392 (9)	
H5A	−0.0180	0.2049	0.1130	0.047*	
C6	0.1082 (3)	0.3222 (3)	0.13611 (19)	0.0315 (7)	
H6A	0.0987	0.2965	0.1806	0.038*	
C7	0.3280 (3)	0.6392 (3)	0.10949 (14)	0.0200 (5)	
C8	0.2342 (3)	0.7205 (3)	0.08113 (15)	0.0219 (5)	
H8A	0.1518	0.6993	0.0901	0.026*	
C9	0.2634 (3)	0.8338 (3)	0.03938 (14)	0.0220 (5)	
C10	0.3862 (3)	0.8684 (3)	0.02664 (16)	0.0259 (6)	
H10A	0.4051	0.9450	−0.0008	0.031*	
C11	0.4792 (3)	0.7879 (3)	0.05508 (16)	0.0280 (6)	
H11A	0.5615	0.8100	0.0467	0.034*	
C12	0.4506 (3)	0.6733 (3)	0.09642 (15)	0.0240 (6)	
H12A	0.5140	0.6194	0.1154	0.029*	
Cl3A	0.2009 (2)	0.7404 (2)	0.34772 (10)	0.0659 (7)	0.671 (3)
Cl3B	−0.1486 (3)	0.5340 (3)	0.29616 (17)	0.0448 (9)	0.329 (3)
C13A	0.1723 (8)	0.5374 (15)	0.2151 (6)	0.0208 (15)	0.671 (3)
C14A	0.2198 (6)	0.6039 (10)	0.2575 (4)	0.0280 (15)	0.671 (3)
H14A	0.3044	0.6184	0.2580	0.034*	0.671 (3)
C15A	0.1417 (7)	0.6491 (6)	0.2994 (3)	0.0332 (11)	0.671 (3)
C16A	0.0163 (7)	0.6236 (7)	0.3013 (3)	0.0423 (15)	0.671 (3)
H16A	−0.0354	0.6506	0.3304	0.051*	0.671 (3)
C17A	−0.0308 (6)	0.5572 (6)	0.2591 (3)	0.0378 (13)	0.671 (3)
H17A	−0.1150	0.5403	0.2597	0.045*	0.671 (3)
C18A	0.0456 (6)	0.5157 (8)	0.2160 (4)	0.0255 (13)	0.671 (3)
H18A	0.0120	0.4728	0.1874	0.031*	0.671 (3)
C13B	0.1952 (15)	0.545 (3)	0.2260 (12)	0.021 (3)	0.329 (3)
C18B	0.2578 (12)	0.6063 (19)	0.2661 (9)	0.027 (3)	0.329 (3)
H18B	0.3423	0.6202	0.2604	0.032*	0.329 (3)
C17B	0.1954 (12)	0.6473 (15)	0.3147 (7)	0.041 (3)	0.329 (3)
H17B	0.2376	0.6883	0.3416	0.049*	0.329 (3)
C16B	0.0685 (12)	0.6259 (10)	0.3225 (6)	0.031 (2)	0.329 (3)
H16B	0.0254	0.6533	0.3546	0.037*	0.329 (3)
C15B	0.0074 (12)	0.5649 (12)	0.2831 (6)	0.0332 (11)	0.329 (3)
C14B	0.0683 (12)	0.5275 (16)	0.2331 (7)	0.026 (3)	0.329 (3)
H14B	0.0246	0.4914	0.2047	0.031*	0.329 (3)
C19	0.8164 (3)	−0.0659 (3)	0.26168 (15)	0.0236 (6)	
C20	0.9393 (3)	−0.0454 (3)	0.24270 (15)	0.0246 (6)	
H20A	0.9911	−0.0011	0.2640	0.030*	
C21	0.9835 (3)	−0.0919 (3)	0.19156 (16)	0.0264 (6)	
C22	0.9099 (4)	−0.1590 (3)	0.15907 (18)	0.0329 (7)	
H22A	0.9420	−0.1900	0.1252	0.039*	
C23	0.7880 (4)	−0.1793 (4)	0.1777 (2)	0.0389 (9)	
H23A	0.7368	−0.2239	0.1563	0.047*	
C24	0.7421 (3)	−0.1331 (3)	0.2284 (2)	0.0364 (8)	
H24A	0.6599	−0.1472	0.2406	0.044*	
Cl5A	0.48872 (18)	−0.43467 (14)	0.39917 (8)	0.0446 (5)	0.628 (3)
C25A	0.6765 (9)	−0.1170 (8)	0.3814 (4)	0.0241 (18)	0.628 (3)
C26A	0.6287 (6)	−0.2256 (5)	0.3696 (2)	0.0277 (10)	0.628 (3)
H26A	0.6469	−0.2486	0.3312	0.033*	0.628 (3)
C27A	0.5526 (6)	−0.3006 (5)	0.4159 (3)	0.0318 (11)	0.628 (3)
C28A	0.5279 (6)	−0.2715 (5)	0.4748 (3)	0.0331 (12)	0.628 (3)
H28A	0.4780	−0.3227	0.5053	0.040*	0.628 (3)
C29A	0.5788 (10)	−0.1652 (9)	0.4867 (4)	0.030 (2)	0.628 (3)
H29A	0.5655	−0.1452	0.5262	0.036*	0.628 (3)
C30A	0.650 (2)	−0.0878 (13)	0.4403 (7)	0.029 (3)	0.628 (3)
H30A	0.6806	−0.0143	0.4485	0.035*	0.628 (3)
Cl5B	0.6595 (4)	−0.4829 (2)	0.44587 (18)	0.0621 (12)	0.372 (3)
C25B	0.6996 (14)	−0.1120 (12)	0.3943 (7)	0.018 (2)	0.372 (3)
C26B	0.7019 (10)	−0.2373 (8)	0.3936 (4)	0.0294 (18)	0.372 (3)
H26B	0.7337	−0.2632	0.3582	0.035*	0.372 (3)
C27B	0.6550 (11)	−0.3260 (8)	0.4475 (5)	0.038 (2)	0.372 (3)
C28B	0.6025 (10)	−0.2900 (9)	0.4995 (5)	0.033 (2)	0.372 (3)
H28B	0.5672	−0.3486	0.5333	0.040*	0.372 (3)
C29B	0.6037 (15)	−0.1649 (11)	0.5003 (6)	0.023 (3)	0.372 (3)
H29B	0.5700	−0.1385	0.5352	0.027*	0.372 (3)
C30B	0.655 (3)	−0.0781 (19)	0.4491 (11)	0.017 (3)	0.372 (3)
H30B	0.6600	0.0051	0.4517	0.021*	0.372 (3)
C31	0.8909 (3)	0.0507 (3)	0.36231 (14)	0.0247 (6)	
C32	0.9627 (3)	−0.0439 (3)	0.40081 (15)	0.0292 (7)	
H32A	0.9388	−0.1272	0.4085	0.035*	
C33	1.0714 (3)	−0.0106 (4)	0.42754 (15)	0.0322 (8)	
C34	1.1090 (3)	0.1116 (4)	0.41755 (16)	0.0334 (7)	
H34A	1.1812	0.1314	0.4365	0.040*	
C35	1.0381 (3)	0.2039 (4)	0.37912 (16)	0.0307 (7)	
H35A	1.0630	0.2868	0.3714	0.037*	
C36	0.9292 (3)	0.1742 (3)	0.35158 (15)	0.0267 (6)	
H36A	0.8818	0.2375	0.3258	0.032*	
C37	0.5301 (3)	0.3744 (3)	0.13088 (15)	0.0228 (6)	
C38	0.5456 (3)	0.4933 (3)	0.23579 (16)	0.0287 (7)	
C39	0.3684 (3)	0.3288 (3)	0.29689 (15)	0.0253 (6)	
C40	0.6417 (3)	0.2529 (3)	0.35371 (16)	0.0266 (6)	
C41	0.7290 (3)	0.2659 (3)	0.22648 (15)	0.0229 (6)	
C42	0.4764 (3)	0.0766 (3)	0.33450 (15)	0.0252 (6)	
C43	0.2945 (3)	0.1317 (3)	0.24028 (15)	0.0255 (6)	
C44	0.3617 (3)	0.1470 (3)	0.11005 (16)	0.0254 (6)	
C45	0.4376 (3)	−0.0606 (3)	0.21284 (19)	0.0321 (7)	
C46	0.6089 (3)	0.1238 (3)	0.15216 (16)	0.0260 (6)	
O1W	0.8593 (5)	0.6098 (5)	0.5004 (3)	0.0412 (15)*	0.523 (7)
H1WA	0.8196	0.6088	0.4662	0.062*	0.523 (7)
H2WA	0.8094	0.6336	0.5263	0.062*	0.523 (7)
O2W	0.9671 (4)	0.5957 (4)	0.5008 (2)	0.0243 (12)*	0.477 (7)
H1WB	1.0286	0.6386	0.5075	0.036*	0.477 (7)
H2WB	0.9504	0.5440	0.5361	0.036*	0.477 (7)

### Atomic displacement parameters (Å^2^)

**Table:**

	*U*^11^	*U*^22^	*U*^33^	*U*^12^	*U*^13^	*U*^23^
Ru1	0.01803 (11)	0.01647 (10)	0.01851 (10)	−0.00076 (8)	−0.00028 (8)	−0.00513 (7)
Ru2	0.01584 (10)	0.01954 (11)	0.02046 (10)	0.00065 (8)	0.00184 (8)	−0.00405 (8)
Ru3	0.01830 (11)	0.01931 (11)	0.02506 (11)	−0.00153 (8)	0.00263 (9)	−0.00940 (8)
Cl1	0.0736 (8)	0.0353 (5)	0.0341 (4)	−0.0104 (5)	−0.0196 (5)	−0.0076 (4)
Cl2	0.0289 (4)	0.0192 (3)	0.0330 (4)	0.0013 (3)	−0.0025 (3)	−0.0021 (3)
Cl4	0.0326 (4)	0.0554 (6)	0.0471 (5)	−0.0144 (4)	0.0175 (4)	−0.0316 (5)
Cl6	0.0415 (5)	0.0516 (6)	0.0298 (4)	0.0216 (4)	−0.0069 (4)	−0.0025 (4)
P1	0.0188 (3)	0.0167 (3)	0.0210 (3)	−0.0002 (3)	0.0027 (3)	−0.0028 (3)
P2	0.0202 (4)	0.0214 (3)	0.0254 (4)	0.0033 (3)	0.0049 (3)	−0.0018 (3)
O1	0.0353 (13)	0.0298 (12)	0.0274 (11)	−0.0022 (10)	0.0082 (10)	−0.0067 (9)
O2	0.062 (2)	0.0402 (16)	0.0511 (17)	−0.0212 (15)	−0.0073 (15)	−0.0202 (13)
O3	0.0331 (13)	0.0405 (14)	0.0241 (11)	0.0031 (11)	0.0047 (10)	−0.0050 (10)
O4	0.0386 (15)	0.0560 (18)	0.0357 (14)	0.0077 (13)	−0.0037 (12)	−0.0242 (13)
O5	0.0246 (12)	0.0399 (14)	0.0282 (11)	−0.0066 (10)	0.0033 (9)	−0.0018 (10)
O6	0.0236 (12)	0.0458 (15)	0.0303 (12)	−0.0030 (11)	0.0036 (10)	0.0022 (11)
O7	0.0215 (11)	0.0441 (14)	0.0294 (11)	−0.0022 (10)	0.0035 (9)	−0.0139 (10)
O8	0.0283 (12)	0.0478 (15)	0.0299 (12)	−0.0024 (11)	0.0008 (10)	−0.0175 (11)
O9	0.0311 (14)	0.0237 (13)	0.097 (3)	−0.0037 (11)	0.0019 (16)	−0.0097 (15)
O10	0.0274 (12)	0.0356 (13)	0.0360 (13)	−0.0001 (10)	0.0065 (10)	−0.0137 (10)
C1	0.0172 (13)	0.0196 (13)	0.0303 (14)	−0.0015 (10)	−0.0037 (11)	−0.0025 (11)
C2	0.0261 (15)	0.0199 (13)	0.0310 (15)	−0.0041 (12)	−0.0059 (12)	−0.0011 (11)
C3	0.0373 (19)	0.0225 (14)	0.0351 (17)	−0.0020 (14)	−0.0130 (15)	−0.0044 (13)
C4	0.0321 (19)	0.0267 (16)	0.058 (2)	−0.0052 (14)	−0.0191 (18)	−0.0043 (16)
C5	0.0213 (16)	0.0271 (16)	0.065 (3)	−0.0073 (13)	−0.0088 (17)	−0.0022 (17)
C6	0.0205 (15)	0.0264 (15)	0.0443 (19)	−0.0029 (12)	−0.0016 (14)	−0.0017 (14)
C7	0.0220 (13)	0.0174 (12)	0.0205 (12)	−0.0027 (10)	0.0022 (10)	−0.0040 (10)
C8	0.0231 (14)	0.0168 (12)	0.0255 (13)	−0.0009 (11)	0.0028 (11)	−0.0046 (10)
C9	0.0252 (14)	0.0177 (12)	0.0232 (13)	−0.0001 (11)	0.0000 (11)	−0.0048 (10)
C10	0.0289 (16)	0.0194 (13)	0.0276 (14)	−0.0060 (12)	0.0011 (12)	−0.0015 (11)
C11	0.0244 (15)	0.0250 (15)	0.0322 (15)	−0.0074 (12)	0.0031 (13)	−0.0015 (12)
C12	0.0217 (14)	0.0217 (13)	0.0279 (14)	−0.0037 (11)	0.0021 (11)	−0.0040 (11)
Cl3A	0.0624 (12)	0.0899 (15)	0.0665 (12)	0.0127 (10)	−0.0044 (9)	−0.0618 (12)
Cl3B	0.0233 (13)	0.0548 (19)	0.059 (2)	0.0030 (12)	0.0115 (12)	−0.0201 (15)
C13A	0.023 (3)	0.017 (3)	0.021 (4)	0.001 (3)	0.001 (3)	−0.003 (2)
C14A	0.020 (3)	0.032 (3)	0.034 (3)	−0.001 (3)	0.002 (3)	−0.012 (2)
C15A	0.036 (3)	0.034 (3)	0.033 (3)	0.008 (2)	0.003 (2)	−0.016 (2)
C16A	0.030 (3)	0.061 (4)	0.040 (3)	0.018 (3)	0.005 (3)	−0.020 (3)
C17A	0.027 (3)	0.043 (3)	0.043 (3)	0.006 (2)	0.006 (2)	−0.011 (3)
C18A	0.020 (3)	0.026 (3)	0.030 (3)	0.003 (2)	0.000 (2)	−0.007 (3)
C13B	0.020 (6)	0.018 (6)	0.025 (9)	−0.002 (6)	0.006 (6)	−0.003 (6)
C18B	0.016 (6)	0.025 (5)	0.046 (8)	−0.001 (6)	0.007 (6)	−0.022 (5)
C17B	0.021 (6)	0.057 (8)	0.053 (8)	−0.002 (6)	0.007 (6)	−0.033 (7)
C16B	0.029 (6)	0.025 (5)	0.042 (7)	0.007 (5)	0.006 (6)	−0.013 (5)
C15B	0.036 (3)	0.034 (3)	0.033 (3)	0.008 (2)	0.003 (2)	−0.016 (2)
C14B	0.023 (6)	0.028 (6)	0.026 (7)	−0.001 (5)	−0.007 (5)	−0.004 (5)
C19	0.0219 (14)	0.0192 (13)	0.0296 (14)	0.0034 (11)	0.0028 (12)	−0.0055 (11)
C20	0.0268 (15)	0.0253 (14)	0.0221 (13)	−0.0013 (12)	0.0033 (11)	−0.0061 (11)
C21	0.0251 (15)	0.0279 (15)	0.0267 (14)	−0.0007 (12)	0.0040 (12)	−0.0071 (12)
C22	0.0363 (19)	0.0311 (17)	0.0349 (17)	0.0039 (15)	−0.0010 (15)	−0.0154 (14)
C23	0.0309 (18)	0.0313 (18)	0.062 (3)	0.0020 (15)	−0.0036 (18)	−0.0267 (18)
C24	0.0203 (15)	0.0295 (17)	0.064 (2)	0.0016 (13)	0.0010 (16)	−0.0195 (17)
Cl5A	0.0634 (11)	0.0292 (7)	0.0399 (8)	−0.0127 (7)	0.0097 (7)	−0.0053 (6)
C25A	0.021 (4)	0.030 (3)	0.020 (3)	0.005 (2)	0.001 (3)	−0.003 (2)
C26A	0.035 (3)	0.025 (2)	0.022 (2)	0.008 (2)	0.004 (2)	−0.0041 (18)
C27A	0.040 (3)	0.024 (2)	0.029 (2)	−0.002 (2)	0.004 (2)	−0.0021 (19)
C28A	0.043 (3)	0.028 (3)	0.025 (2)	0.001 (2)	0.010 (2)	0.0014 (19)
C29A	0.036 (5)	0.038 (3)	0.015 (3)	0.004 (3)	0.000 (3)	−0.003 (2)
C30A	0.033 (5)	0.033 (4)	0.021 (4)	0.000 (4)	−0.005 (4)	−0.005 (3)
Cl5B	0.092 (3)	0.0191 (11)	0.074 (2)	−0.0119 (13)	0.037 (2)	−0.0100 (12)
C25B	0.012 (5)	0.019 (4)	0.023 (6)	0.000 (3)	−0.001 (4)	−0.004 (3)
C26B	0.034 (5)	0.025 (4)	0.030 (4)	−0.002 (4)	0.008 (4)	−0.008 (3)
C27B	0.047 (6)	0.020 (3)	0.047 (5)	−0.010 (4)	0.022 (5)	−0.007 (3)
C28B	0.039 (5)	0.028 (4)	0.032 (4)	−0.009 (4)	0.010 (4)	−0.003 (3)
C29B	0.028 (6)	0.021 (4)	0.018 (6)	−0.008 (4)	−0.001 (4)	−0.002 (3)
C30B	0.019 (5)	0.020 (4)	0.014 (6)	−0.005 (4)	−0.002 (5)	−0.008 (3)
C31	0.0232 (14)	0.0313 (15)	0.0190 (12)	0.0069 (12)	0.0046 (11)	−0.0051 (11)
C32	0.0314 (17)	0.0344 (17)	0.0206 (13)	0.0096 (14)	0.0046 (12)	−0.0044 (12)
C33	0.0270 (16)	0.047 (2)	0.0196 (13)	0.0177 (15)	0.0001 (12)	−0.0037 (13)
C34	0.0246 (16)	0.050 (2)	0.0246 (15)	0.0071 (15)	0.0010 (13)	−0.0071 (14)
C35	0.0239 (15)	0.0414 (19)	0.0265 (15)	0.0022 (14)	−0.0003 (12)	−0.0071 (13)
C36	0.0219 (14)	0.0328 (16)	0.0236 (13)	0.0067 (13)	0.0020 (11)	−0.0034 (12)
C37	0.0218 (14)	0.0211 (13)	0.0258 (13)	−0.0004 (11)	−0.0006 (11)	−0.0059 (11)
C38	0.0321 (17)	0.0279 (15)	0.0278 (15)	−0.0045 (13)	−0.0018 (13)	−0.0098 (12)
C39	0.0252 (15)	0.0237 (14)	0.0258 (14)	0.0012 (12)	−0.0029 (12)	−0.0034 (11)
C40	0.0205 (14)	0.0320 (16)	0.0282 (14)	0.0038 (12)	−0.0014 (12)	−0.0092 (12)
C41	0.0191 (13)	0.0265 (14)	0.0231 (13)	0.0003 (11)	−0.0019 (11)	−0.0058 (11)
C42	0.0205 (14)	0.0301 (15)	0.0226 (13)	0.0010 (12)	0.0012 (11)	−0.0015 (11)
C43	0.0242 (15)	0.0291 (15)	0.0249 (13)	−0.0002 (12)	−0.0010 (12)	−0.0099 (12)
C44	0.0189 (13)	0.0277 (15)	0.0330 (15)	−0.0039 (12)	0.0053 (12)	−0.0140 (12)
C45	0.0224 (15)	0.0273 (16)	0.048 (2)	−0.0038 (13)	0.0047 (14)	−0.0109 (14)
C46	0.0257 (15)	0.0237 (14)	0.0305 (15)	−0.0015 (12)	0.0022 (12)	−0.0102 (12)

### Geometric parameters (Å, °)

**Table:**

Ru1—C38	1.885 (3)	C15A—C16A	1.385 (10)
Ru1—C37	1.939 (3)	C16A—C17A	1.384 (10)
Ru1—C39	1.940 (3)	C16A—H16A	0.9300
Ru1—P1	2.3436 (8)	C17A—C18A	1.383 (8)
Ru1—Ru2	2.8517 (3)	C17A—H17A	0.9300
Ru1—Ru3	2.8645 (3)	C18A—H18A	0.9300
Ru2—C40	1.889 (3)	C13B—C14B	1.389 (14)
Ru2—C42	1.934 (3)	C13B—C18B	1.389 (14)
Ru2—C41	1.939 (3)	C18B—C17B	1.391 (13)
Ru2—P2	2.3423 (8)	C18B—H18B	0.9300
Ru2—Ru3	2.8634 (3)	C17B—C16B	1.397 (14)
Ru3—C45	1.908 (4)	C17B—H17B	0.9300
Ru3—C44	1.920 (3)	C16B—C15B	1.366 (15)
Ru3—C43	1.945 (3)	C16B—H16B	0.9300
Ru3—C46	1.956 (3)	C15B—C14B	1.389 (13)
Cl1—C3	1.743 (4)	C14B—H14B	0.9300
Cl2—C9	1.739 (3)	C19—C20	1.392 (4)
Cl4—C21	1.725 (3)	C19—C24	1.398 (5)
Cl6—C33	1.745 (4)	C20—C21	1.388 (4)
P1—C13A	1.829 (9)	C20—H20A	0.9300
P1—C1	1.833 (3)	C21—C22	1.382 (5)
P1—C7	1.840 (3)	C22—C23	1.380 (5)
P1—C13B	1.889 (16)	C22—H22A	0.9300
P2—C25A	1.821 (9)	C23—C24	1.384 (6)
P2—C19	1.834 (3)	C23—H23A	0.9300
P2—C31	1.842 (3)	C24—H24A	0.9300
P2—C25B	1.855 (14)	Cl5A—C27A	1.743 (6)
O1—C37	1.137 (4)	C25A—C26A	1.381 (9)
O2—C38	1.149 (4)	C25A—C30A	1.393 (9)
O3—C39	1.140 (4)	C26A—C27A	1.399 (7)
O4—C40	1.141 (4)	C26A—H26A	0.9300
O5—C41	1.144 (4)	C27A—C28A	1.390 (7)
O6—C42	1.141 (4)	C28A—C29A	1.372 (9)
O7—C43	1.145 (4)	C28A—H28A	0.9300
O8—C44	1.139 (4)	C29A—C30A	1.380 (10)
O9—C45	1.145 (4)	C29A—H29A	0.9300
O10—C46	1.136 (4)	C30A—H30A	0.9300
C1—C2	1.394 (5)	Cl5B—C27B	1.731 (8)
C1—C6	1.406 (4)	C25B—C26B	1.380 (13)
C2—C3	1.387 (5)	C25B—C30B	1.388 (13)
C2—H2A	0.9300	C26B—C27B	1.418 (11)
C3—C4	1.373 (5)	C26B—H26B	0.9300
C4—C5	1.384 (6)	C27B—C28B	1.380 (11)
C4—H4A	0.9300	C28B—C29B	1.378 (12)
C5—C6	1.396 (5)	C28B—H28B	0.9300
C5—H5A	0.9300	C29B—C30B	1.389 (12)
C6—H6A	0.9300	C29B—H29B	0.9300
C7—C8	1.388 (4)	C30B—H30B	0.9300
C7—C12	1.390 (4)	C31—C36	1.389 (5)
C8—C9	1.389 (4)	C31—C32	1.395 (5)
C8—H8A	0.9300	C32—C33	1.396 (5)
C9—C10	1.392 (4)	C32—H32A	0.9300
C10—C11	1.377 (5)	C33—C34	1.374 (6)
C10—H10A	0.9300	C34—C35	1.374 (5)
C11—C12	1.395 (4)	C34—H34A	0.9300
C11—H11A	0.9300	C35—C36	1.392 (5)
C12—H12A	0.9300	C35—H35A	0.9300
Cl3A—C15A	1.725 (7)	C36—H36A	0.9300
Cl3B—C15B	1.732 (13)	O1W—H1WA	0.8499
C13A—C14A	1.391 (9)	O1W—H2WA	0.8500
C13A—C18A	1.392 (9)	O2W—H1WB	0.8499
C14A—C15A	1.394 (8)	O2W—H2WB	0.8501
C14A—H14A	0.9300		
			
C38—Ru1—C37	96.15 (14)	C18A—C17A—C16A	121.0 (6)
C38—Ru1—C39	92.10 (14)	C18A—C17A—H17A	119.5
C37—Ru1—C39	171.10 (13)	C16A—C17A—H17A	119.5
C38—Ru1—P1	98.22 (11)	C17A—C18A—C13A	120.6 (7)
C37—Ru1—P1	89.65 (10)	C17A—C18A—H18A	119.7
C39—Ru1—P1	92.48 (10)	C13A—C18A—H18A	119.7
C38—Ru1—Ru2	93.03 (11)	C14B—C13B—C18B	119.6 (12)
C37—Ru1—Ru2	96.75 (9)	C14B—C13B—P1	122.2 (10)
C39—Ru1—Ru2	79.43 (10)	C18B—C13B—P1	118.2 (10)
P1—Ru1—Ru2	166.40 (2)	C13B—C18B—C17B	120.7 (12)
C38—Ru1—Ru3	148.79 (11)	C13B—C18B—H18B	119.7
C37—Ru1—Ru3	73.58 (9)	C17B—C18B—H18B	119.7
C39—Ru1—Ru3	97.60 (10)	C18B—C17B—C16B	119.0 (11)
P1—Ru1—Ru3	110.84 (2)	C18B—C17B—H17B	120.5
Ru2—Ru1—Ru3	60.123 (8)	C16B—C17B—H17B	120.5
C40—Ru2—C42	91.90 (14)	C15B—C16B—C17B	120.1 (10)
C40—Ru2—C41	95.74 (14)	C15B—C16B—H16B	119.9
C42—Ru2—C41	171.32 (13)	C17B—C16B—H16B	119.9
C40—Ru2—P2	94.52 (10)	C16B—C15B—C14B	121.1 (11)
C42—Ru2—P2	91.48 (10)	C16B—C15B—Cl3B	119.0 (9)
C41—Ru2—P2	92.00 (10)	C14B—C15B—Cl3B	119.8 (10)
C40—Ru2—Ru1	95.96 (10)	C15B—C14B—C13B	119.3 (12)
C42—Ru2—Ru1	95.18 (10)	C15B—C14B—H14B	120.4
C41—Ru2—Ru1	79.98 (9)	C13B—C14B—H14B	120.4
P2—Ru2—Ru1	167.38 (2)	C20—C19—C24	118.5 (3)
C40—Ru2—Ru3	150.18 (10)	C20—C19—P2	119.9 (2)
C42—Ru2—Ru3	74.29 (10)	C24—C19—P2	121.3 (3)
C41—Ru2—Ru3	97.03 (9)	C21—C20—C19	119.1 (3)
P2—Ru2—Ru3	111.81 (2)	C21—C20—H20A	120.5
Ru1—Ru2—Ru3	60.160 (8)	C19—C20—H20A	120.5
C45—Ru3—C44	97.84 (15)	C22—C21—C20	122.2 (3)
C45—Ru3—C43	92.08 (15)	C22—C21—Cl4	119.0 (3)
C44—Ru3—C43	94.26 (13)	C20—C21—Cl4	118.8 (3)
C45—Ru3—C46	94.45 (14)	C23—C22—C21	118.9 (3)
C44—Ru3—C46	93.01 (14)	C23—C22—H22A	120.6
C43—Ru3—C46	169.48 (13)	C21—C22—H22A	120.6
C45—Ru3—Ru2	103.74 (12)	C22—C23—C24	119.7 (3)
C44—Ru3—Ru2	156.04 (10)	C22—C23—H23A	120.2
C43—Ru3—Ru2	95.24 (9)	C24—C23—H23A	120.2
C46—Ru3—Ru2	75.20 (10)	C23—C24—C19	121.7 (3)
C45—Ru3—Ru1	157.41 (12)	C23—C24—H24A	119.2
C44—Ru3—Ru1	101.81 (10)	C19—C24—H24A	119.2
C43—Ru3—Ru1	75.54 (10)	C26A—C25A—C30A	118.4 (8)
C46—Ru3—Ru1	95.52 (9)	C26A—C25A—P2	126.7 (6)
Ru2—Ru3—Ru1	59.717 (8)	C30A—C25A—P2	114.0 (7)
C13A—P1—C1	102.7 (3)	C25A—C26A—C27A	119.4 (6)
C13A—P1—C7	100.7 (5)	C25A—C26A—H26A	120.3
C1—P1—C7	102.03 (14)	C27A—C26A—H26A	120.3
C1—P1—C13B	113.5 (5)	C28A—C27A—C26A	121.7 (5)
C7—P1—C13B	99.5 (10)	C28A—C27A—Cl5A	119.5 (4)
C13A—P1—Ru1	116.5 (4)	C26A—C27A—Cl5A	118.9 (4)
C1—P1—Ru1	116.30 (10)	C29A—C28A—C27A	118.4 (6)
C7—P1—Ru1	116.19 (10)	C29A—C28A—H28A	120.8
C13B—P1—Ru1	108.1 (9)	C27A—C28A—H28A	120.8
C25A—P2—C19	105.2 (3)	C28A—C29A—C30A	120.4 (8)
C25A—P2—C31	106.9 (3)	C28A—C29A—H29A	119.8
C19—P2—C31	102.30 (14)	C30A—C29A—H29A	119.8
C19—P2—C25B	110.2 (5)	C29A—C30A—C25A	121.7 (9)
C31—P2—C25B	95.0 (4)	C29A—C30A—H30A	119.2
C25A—P2—Ru2	109.8 (3)	C25A—C30A—H30A	119.2
C19—P2—Ru2	116.10 (11)	C26B—C25B—C30B	118.4 (12)
C31—P2—Ru2	115.63 (11)	C26B—C25B—P2	120.9 (10)
C25B—P2—Ru2	115.1 (5)	C30B—C25B—P2	120.7 (11)
C2—C1—C6	119.2 (3)	C25B—C26B—C27B	119.2 (9)
C2—C1—P1	120.3 (2)	C25B—C26B—H26B	120.4
C6—C1—P1	120.2 (3)	C27B—C26B—H26B	120.4
C3—C2—C1	119.1 (3)	C28B—C27B—C26B	121.7 (8)
C3—C2—H2A	120.5	C28B—C27B—Cl5B	119.7 (7)
C1—C2—H2A	120.5	C26B—C27B—Cl5B	118.5 (7)
C4—C3—C2	122.3 (4)	C29B—C28B—C27B	118.3 (9)
C4—C3—Cl1	118.5 (3)	C29B—C28B—H28B	120.8
C2—C3—Cl1	119.2 (3)	C27B—C28B—H28B	120.8
C3—C4—C5	118.9 (3)	C28B—C29B—C30B	120.2 (13)
C3—C4—H4A	120.6	C28B—C29B—H29B	119.9
C5—C4—H4A	120.6	C30B—C29B—H29B	119.9
C4—C5—C6	120.5 (3)	C25B—C30B—C29B	121.9 (14)
C4—C5—H5A	119.7	C25B—C30B—H30B	119.0
C6—C5—H5A	119.7	C29B—C30B—H30B	119.0
C5—C6—C1	119.9 (4)	C36—C31—C32	119.5 (3)
C5—C6—H6A	120.1	C36—C31—P2	121.1 (2)
C1—C6—H6A	120.1	C32—C31—P2	119.3 (3)
C8—C7—C12	119.3 (3)	C31—C32—C33	118.4 (3)
C8—C7—P1	119.2 (2)	C31—C32—H32A	120.8
C12—C7—P1	121.5 (2)	C33—C32—H32A	120.8
C7—C8—C9	119.9 (3)	C34—C33—C32	122.3 (3)
C7—C8—H8A	120.0	C34—C33—Cl6	118.9 (3)
C9—C8—H8A	120.0	C32—C33—Cl6	118.8 (3)
C8—C9—C10	120.8 (3)	C35—C34—C33	118.8 (3)
C8—C9—Cl2	120.5 (2)	C35—C34—H34A	120.6
C10—C9—Cl2	118.7 (2)	C33—C34—H34A	120.6
C11—C10—C9	119.3 (3)	C34—C35—C36	120.5 (4)
C11—C10—H10A	120.4	C34—C35—H35A	119.7
C9—C10—H10A	120.4	C36—C35—H35A	119.7
C10—C11—C12	120.3 (3)	C31—C36—C35	120.5 (3)
C10—C11—H11A	119.9	C31—C36—H36A	119.8
C12—C11—H11A	119.9	C35—C36—H36A	119.8
C7—C12—C11	120.4 (3)	O1—C37—Ru1	173.6 (3)
C7—C12—H12A	119.8	O2—C38—Ru1	177.7 (3)
C11—C12—H12A	119.8	O3—C39—Ru1	175.2 (3)
C14A—C13A—C18A	118.5 (7)	O4—C40—Ru2	176.0 (3)
C14A—C13A—P1	114.8 (6)	O5—C41—Ru2	175.4 (3)
C18A—C13A—P1	126.7 (6)	O6—C42—Ru2	173.6 (3)
C13A—C14A—C15A	120.6 (7)	O7—C43—Ru3	172.2 (3)
C13A—C14A—H14A	119.7	O8—C44—Ru3	174.8 (3)
C15A—C14A—H14A	119.7	O9—C45—Ru3	176.7 (3)
C16A—C15A—C14A	120.4 (6)	O10—C46—Ru3	173.0 (3)
C16A—C15A—Cl3A	119.7 (5)	H1WA—O1W—H2WA	107.7
C14A—C15A—Cl3A	119.8 (6)	H1WA—O1W—H2WB	138.0
C17A—C16A—C15A	118.9 (5)	H2WA—O1W—H2WB	107.3
C17A—C16A—H16A	120.6	H1WB—O2W—H2WB	107.7
C15A—C16A—H16A	120.6		
			
C38—Ru1—Ru2—C40	35.75 (15)	C2—C1—C6—C5	−2.6 (5)
C37—Ru1—Ru2—C40	132.31 (14)	P1—C1—C6—C5	−177.3 (3)
C39—Ru1—Ru2—C40	−55.82 (14)	C13A—P1—C7—C8	−48.6 (4)
P1—Ru1—Ru2—C40	−110.08 (14)	C1—P1—C7—C8	57.0 (3)
Ru3—Ru1—Ru2—C40	−161.03 (10)	C13B—P1—C7—C8	−59.7 (6)
C38—Ru1—Ru2—C42	128.22 (15)	Ru1—P1—C7—C8	−175.4 (2)
C37—Ru1—Ru2—C42	−135.22 (13)	C13A—P1—C7—C12	132.2 (4)
C39—Ru1—Ru2—C42	36.65 (14)	C1—P1—C7—C12	−122.2 (3)
P1—Ru1—Ru2—C42	−17.62 (13)	C13B—P1—C7—C12	121.1 (6)
Ru3—Ru1—Ru2—C42	−68.57 (10)	Ru1—P1—C7—C12	5.4 (3)
C38—Ru1—Ru2—C41	−59.05 (14)	C12—C7—C8—C9	1.0 (4)
C37—Ru1—Ru2—C41	37.51 (13)	P1—C7—C8—C9	−178.2 (2)
C39—Ru1—Ru2—C41	−150.62 (14)	C7—C8—C9—C10	−1.5 (5)
P1—Ru1—Ru2—C41	155.11 (13)	C7—C8—C9—Cl2	179.5 (2)
Ru3—Ru1—Ru2—C41	104.17 (9)	C8—C9—C10—C11	1.1 (5)
C38—Ru1—Ru2—P2	−110.22 (15)	Cl2—C9—C10—C11	−179.9 (3)
C37—Ru1—Ru2—P2	−13.66 (14)	C9—C10—C11—C12	−0.3 (5)
C39—Ru1—Ru2—P2	158.21 (14)	C8—C7—C12—C11	−0.2 (5)
P1—Ru1—Ru2—P2	103.95 (14)	P1—C7—C12—C11	179.0 (3)
Ru3—Ru1—Ru2—P2	53.00 (11)	C10—C11—C12—C7	−0.2 (5)
C38—Ru1—Ru2—Ru3	−163.22 (11)	C1—P1—C13A—C14A	−174.9 (10)
C37—Ru1—Ru2—Ru3	−66.66 (9)	C7—P1—C13A—C14A	−69.9 (11)
C39—Ru1—Ru2—Ru3	105.21 (10)	C13B—P1—C13A—C14A	15 (7)
P1—Ru1—Ru2—Ru3	50.95 (9)	Ru1—P1—C13A—C14A	56.7 (12)
C40—Ru2—Ru3—C45	−122.8 (2)	C1—P1—C13A—C18A	4.8 (15)
C42—Ru2—Ru3—C45	−57.69 (15)	C7—P1—C13A—C18A	109.9 (13)
C41—Ru2—Ru3—C45	122.52 (15)	C13B—P1—C13A—C18A	−166 (10)
P2—Ru2—Ru3—C45	27.51 (11)	Ru1—P1—C13A—C18A	−123.5 (12)
Ru1—Ru2—Ru3—C45	−163.32 (11)	C18A—C13A—C14A—C15A	−0.8 (19)
C40—Ru2—Ru3—C44	83.6 (3)	P1—C13A—C14A—C15A	178.9 (9)
C42—Ru2—Ru3—C44	148.7 (3)	C13A—C14A—C15A—C16A	2.9 (14)
C41—Ru2—Ru3—C44	−31.1 (3)	C13A—C14A—C15A—Cl3A	−175.6 (10)
P2—Ru2—Ru3—C44	−126.1 (2)	C14A—C15A—C16A—C17A	−2.7 (11)
Ru1—Ru2—Ru3—C44	43.1 (2)	Cl3A—C15A—C16A—C17A	175.8 (5)
C40—Ru2—Ru3—C43	−29.3 (2)	C15A—C16A—C17A—C18A	0.6 (11)
C42—Ru2—Ru3—C43	35.75 (14)	C16A—C17A—C18A—C13A	1.4 (14)
C41—Ru2—Ru3—C43	−144.05 (14)	C14A—C13A—C18A—C17A	−1.3 (18)
P2—Ru2—Ru3—C43	120.95 (10)	P1—C13A—C18A—C17A	179.0 (9)
Ru1—Ru2—Ru3—C43	−69.89 (10)	C13A—P1—C13B—C14B	14 (6)
C40—Ru2—Ru3—C46	146.2 (2)	C1—P1—C13B—C14B	4(3)
C42—Ru2—Ru3—C46	−148.73 (14)	C7—P1—C13B—C14B	111 (3)
C41—Ru2—Ru3—C46	31.48 (14)	Ru1—P1—C13B—C14B	−127 (2)
P2—Ru2—Ru3—C46	−63.53 (10)	C13A—P1—C13B—C18B	−166 (10)
Ru1—Ru2—Ru3—C46	105.64 (10)	C1—P1—C13B—C18B	−176 (2)
C40—Ru2—Ru3—Ru1	40.5 (2)	C7—P1—C13B—C18B	−68 (2)
C42—Ru2—Ru3—Ru1	105.63 (10)	Ru1—P1—C13B—C18B	53 (3)
C41—Ru2—Ru3—Ru1	−74.16 (10)	C14B—C13B—C18B—C17B	2(4)
P2—Ru2—Ru3—Ru1	−169.16 (2)	P1—C13B—C18B—C17B	−178.2 (19)
C38—Ru1—Ru3—C45	80.4 (3)	C13B—C18B—C17B—C16B	0(3)
C37—Ru1—Ru3—C45	154.6 (3)	C18B—C17B—C16B—C15B	0(2)
C39—Ru1—Ru3—C45	−26.6 (3)	C17B—C16B—C15B—C14B	−3(2)
P1—Ru1—Ru3—C45	−122.2 (3)	C17B—C16B—C15B—Cl3B	177.3 (11)
Ru2—Ru1—Ru3—C45	46.5 (3)	C16B—C15B—C14B—C13B	5(3)
C38—Ru1—Ru3—C44	−129.7 (2)	Cl3B—C15B—C14B—C13B	−175 (2)
C37—Ru1—Ru3—C44	−55.45 (13)	C18B—C13B—C14B—C15B	−4(4)
C39—Ru1—Ru3—C44	123.32 (14)	P1—C13B—C14B—C15B	176.0 (17)
P1—Ru1—Ru3—C44	27.73 (10)	C25A—P2—C19—C20	−130.3 (4)
Ru2—Ru1—Ru3—C44	−163.54 (9)	C31—P2—C19—C20	−18.7 (3)
C38—Ru1—Ru3—C43	138.9 (2)	C25B—P2—C19—C20	−118.8 (5)
C37—Ru1—Ru3—C43	−146.85 (14)	Ru2—P2—C19—C20	108.1 (3)
C39—Ru1—Ru3—C43	31.92 (14)	C25A—P2—C19—C24	56.3 (4)
P1—Ru1—Ru3—C43	−63.67 (10)	C31—P2—C19—C24	167.8 (3)
Ru2—Ru1—Ru3—C43	105.06 (10)	C25B—P2—C19—C24	67.8 (6)
C38—Ru1—Ru3—C46	−35.5 (2)	Ru2—P2—C19—C24	−65.3 (3)
C37—Ru1—Ru3—C46	38.80 (14)	C24—C19—C20—C21	0.2 (5)
C39—Ru1—Ru3—C46	−142.43 (14)	P2—C19—C20—C21	−173.4 (2)
P1—Ru1—Ru3—C46	121.98 (10)	C19—C20—C21—C22	−0.6 (5)
Ru2—Ru1—Ru3—C46	−69.29 (10)	C19—C20—C21—Cl4	−179.2 (2)
C38—Ru1—Ru3—Ru2	33.8 (2)	C20—C21—C22—C23	0.7 (6)
C37—Ru1—Ru3—Ru2	108.09 (10)	Cl4—C21—C22—C23	179.3 (3)
C39—Ru1—Ru3—Ru2	−73.14 (10)	C21—C22—C23—C24	−0.4 (6)
P1—Ru1—Ru3—Ru2	−168.73 (2)	C22—C23—C24—C19	0.0 (6)
C38—Ru1—P1—C13A	−77.2 (5)	C20—C19—C24—C23	0.1 (6)
C37—Ru1—P1—C13A	−173.4 (5)	P2—C19—C24—C23	173.6 (3)
C39—Ru1—P1—C13A	15.2 (5)	C19—P2—C25A—C26A	−26.8 (9)
Ru2—Ru1—P1—C13A	68.3 (5)	C31—P2—C25A—C26A	−135.0 (8)
Ru3—Ru1—P1—C13A	114.4 (4)	C25B—P2—C25A—C26A	−143 (4)
C38—Ru1—P1—C1	161.40 (16)	Ru2—P2—C25A—C26A	98.8 (8)
C37—Ru1—P1—C1	65.24 (15)	C19—P2—C25A—C30A	163.8 (13)
C39—Ru1—P1—C1	−106.12 (15)	C31—P2—C25A—C30A	55.5 (14)
Ru2—Ru1—P1—C1	−53.11 (16)	C25B—P2—C25A—C30A	47 (4)
Ru3—Ru1—P1—C1	−7.02 (12)	Ru2—P2—C25A—C30A	−70.6 (14)
C38—Ru1—P1—C7	41.25 (15)	C30A—C25A—C26A—C27A	1.8 (17)
C37—Ru1—P1—C7	−54.91 (14)	P2—C25A—C26A—C27A	−167.2 (6)
C39—Ru1—P1—C7	133.73 (14)	C25A—C26A—C27A—C28A	−2.7 (10)
Ru2—Ru1—P1—C7	−173.26 (11)	C25A—C26A—C27A—Cl5A	177.9 (6)
Ru3—Ru1—P1—C7	−127.17 (10)	C26A—C27A—C28A—C29A	0.9 (10)
C38—Ru1—P1—C13B	−69.5 (8)	Cl5A—C27A—C28A—C29A	−179.8 (6)
C37—Ru1—P1—C13B	−165.7 (8)	C27A—C28A—C29A—C30A	1.9 (18)
C39—Ru1—P1—C13B	23.0 (8)	C28A—C29A—C30A—C25A	−3(3)
Ru2—Ru1—P1—C13B	76.0 (8)	C26A—C25A—C30A—C29A	1(3)
Ru3—Ru1—P1—C13B	122.1 (8)	P2—C25A—C30A—C29A	171.2 (16)
C40—Ru2—P2—C25A	87.4 (3)	C25A—P2—C25B—C26B	61 (3)
C42—Ru2—P2—C25A	−4.7 (3)	C19—P2—C25B—C26B	−6.2 (12)
C41—Ru2—P2—C25A	−176.7 (3)	C31—P2—C25B—C26B	−111.3 (11)
Ru1—Ru2—P2—C25A	−126.6 (3)	Ru2—P2—C25B—C26B	127.4 (10)
Ru3—Ru2—P2—C25A	−78.3 (3)	C25A—P2—C25B—C30B	−118 (5)
C40—Ru2—P2—C19	−153.54 (16)	C19—P2—C25B—C30B	175 (2)
C42—Ru2—P2—C19	114.43 (15)	C31—P2—C25B—C30B	70 (2)
C41—Ru2—P2—C19	−57.62 (15)	Ru2—P2—C25B—C30B	−51 (2)
Ru1—Ru2—P2—C19	−7.48 (18)	C30B—C25B—C26B—C27B	2(3)
Ru3—Ru2—P2—C19	40.77 (12)	P2—C25B—C26B—C27B	−176.3 (9)
C40—Ru2—P2—C31	−33.67 (15)	C25B—C26B—C27B—C28B	2.7 (18)
C42—Ru2—P2—C31	−125.70 (15)	C25B—C26B—C27B—Cl5B	−179.6 (10)
C41—Ru2—P2—C31	62.25 (14)	C26B—C27B—C28B—C29B	−4.3 (19)
Ru1—Ru2—P2—C31	112.39 (14)	Cl5B—C27B—C28B—C29B	178.0 (10)
Ru3—Ru2—P2—C31	160.64 (11)	C27B—C28B—C29B—C30B	1(3)
C40—Ru2—P2—C25B	75.7 (4)	C26B—C25B—C30B—C29B	−6(4)
C42—Ru2—P2—C25B	−16.4 (4)	P2—C25B—C30B—C29B	173 (2)
C41—Ru2—P2—C25B	171.6 (4)	C28B—C29B—C30B—C25B	4(4)
Ru1—Ru2—P2—C25B	−138.3 (4)	C25A—P2—C31—C36	−143.2 (4)
Ru3—Ru2—P2—C25B	−90.0 (4)	C19—P2—C31—C36	106.5 (3)
C13A—P1—C1—C2	130.9 (6)	C25B—P2—C31—C36	−141.5 (5)
C7—P1—C1—C2	26.8 (3)	Ru2—P2—C31—C36	−20.6 (3)
C13B—P1—C1—C2	132.9 (12)	C25A—P2—C31—C32	40.0 (4)
Ru1—P1—C1—C2	−100.7 (3)	C19—P2—C31—C32	−70.3 (3)
C13A—P1—C1—C6	−54.5 (6)	C25B—P2—C31—C32	41.7 (5)
C7—P1—C1—C6	−158.5 (3)	Ru2—P2—C31—C32	162.6 (2)
C13B—P1—C1—C6	−52.5 (12)	C36—C31—C32—C33	0.2 (4)
Ru1—P1—C1—C6	74.0 (3)	P2—C31—C32—C33	177.0 (2)
C6—C1—C2—C3	0.4 (5)	C31—C32—C33—C34	0.5 (5)
P1—C1—C2—C3	175.1 (3)	C31—C32—C33—Cl6	179.9 (2)
C1—C2—C3—C4	1.9 (6)	C32—C33—C34—C35	−1.0 (5)
C1—C2—C3—Cl1	−177.6 (3)	Cl6—C33—C34—C35	179.5 (3)
C2—C3—C4—C5	−2.0 (6)	C33—C34—C35—C36	0.9 (5)
Cl1—C3—C4—C5	177.5 (3)	C32—C31—C36—C35	−0.2 (5)
C3—C4—C5—C6	−0.3 (6)	P2—C31—C36—C35	−177.0 (2)
C4—C5—C6—C1	2.5 (6)	C34—C35—C36—C31	−0.3 (5)

### Hydrogen-bond geometry (Å, °)

**Table:**

*D*—H···*A*	*D*—H	H···*A*	*D*···*A*	*D*—H···*A*
C4—H4A···Cl2^i^	0.93	2.81	3.607 (4)	145
C6—H6A···O7	0.93	2.55	3.259 (5)	134
C11—H11A···O8^ii^	0.93	2.57	3.234 (4)	129
C24—H24A···O9	0.93	2.55	3.425 (5)	157

Symmetry codes: (i) −*x*, −*y*+1, −*z*; (ii) −*x*+1, −*y*+1, −*z*.
